# The script concordance test in radiation oncology: validation study of a new tool to assess clinical reasoning

**DOI:** 10.1186/1748-717X-4-7

**Published:** 2009-02-09

**Authors:** Carole Lambert, Robert Gagnon, David Nguyen, Bernard Charlin

**Affiliations:** 1 CPASS, Faculty of Medicine, University of Montreal, CP 6128, Succursale Centre-Ville, Montreal, Quebec, H3C 3J7, Canada; 2 CHUM (Centre Hospitalier de l'Université de Montréal), Notre-Dame Hospital, radiation oncology department, 1560 Sherbrooke East, Montreal, Quebec, H2L 4M1, Canada

## Abstract

**Background:**

The Script Concordance test (SCT) is a reliable and valid tool to evaluate clinical reasoning in complex situations where experts' opinions may be divided. Scores reflect the degree of concordance between the performance of examinees and that of a reference panel of experienced physicians. The purpose of this study is to demonstrate SCT's usefulness in radiation oncology.

**Methods:**

A 90 items radiation oncology SCT was administered to 155 participants. Three levels of experience were tested: medical students (n = 70), radiation oncology residents (n = 38) and radiation oncologists (n = 47). Statistical tests were performed to assess reliability and to document validity.

**Results:**

After item optimization, the test comprised 30 cases and 70 questions. Cronbach alpha was 0.90. Mean scores were 51.62 (± 8.19) for students, 71.20 (± 9.45) for residents and 76.67 (± 6.14) for radiation oncologists. The difference between the three groups was statistically significant when compared by the Kruskall-Wallis test (p < 0.001).

**Conclusion:**

The SCT is reliable and useful to discriminate among participants according to their level of experience in radiation oncology. It appears as a useful tool to document the progression of reasoning during residency training.

## Background

In oncology, a constant flow of new data from research exposes the physician to an abundance of treatment alternatives [1-3]. The clinician is often challenged by ill-defined problems [4,5] characterized by uncertainty, and opinions on the treatment of a particular patient may differ considerably [6]. Reasoning on treatment options should be monitored to provide information on residents' strengths and weaknesses and to guide their learning.

Experienced practitioners possess elaborate networks of knowledge, called scripts [7] fitted to adapt to their clinical tasks. Scripts allow the clinician to determine diagnosis, strategies of investigation, or treatment options. Scripts begin to appear during medical school and are refined over years of clinical experience [8]. The script concordance test (SCT), which is based on cognitive psychology script theory [9], provides a way to assess reasoning skills in the context of uncertainty that often characterizes oncology.

SCT makes it possible to include real-life situations that are scarcely ever measured with usual tests. It probes the multiple judgments that are made in the clinical reasoning process. Scoring reflects the degree of concordance of these judgments to those of a panel of reference. A series of studies, held in domains such as intra-operative decision-making skills [10], urology [11] or family medicine [12,13] documents the reliability and construct validity of test scores.

Research questions were

1- Is it possible to obtain reliable scores on clinical reasoning in radiation oncology?

2- Do SCT scores reflect participants' level of clinical experience?

3- How the test is perceived by residents and experienced professionals?

## Methods

### Instrument

**Figure 1:**
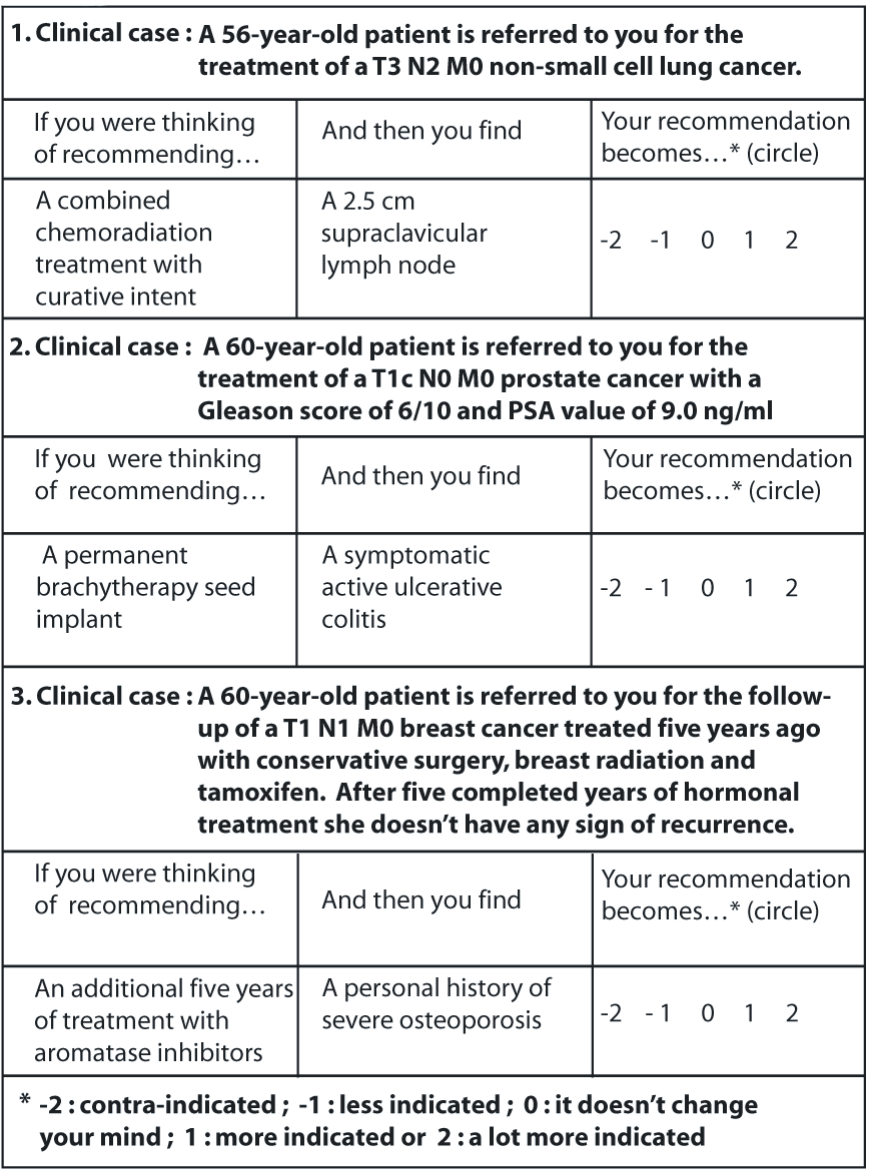
**Items from the pulmonary, urological and breast cancer portions of the test**.

SCTs [9] are made up of cases that incorporate the uncertainty of practice situations. Several options are relevant in solving the diagnostic or management problem posed by the situation. Case scenarios are followed by a series of questions, presented in three parts (See Figure 1). The first part ("if you were thinking of") contains a relevant option. The second part ("and then you find") presents a new clinical finding, such as a physical sign, a pre-existing condition, an imaging study or a laboratory test result. The third part ("this option becomes") is a five-point Likert scale that captures the examinees' decision. The task for examinees is to decide what effect the new finding has in direction (positive, negative or neutral) and intensity, on the status of the option. This effect is captured with a Likert scale because script theory assumes that clinical reasoning is composed of a series of qualitative judgments [7]. The radio-oncology test was constructed by two radiation oncologists. Cases were taken from the three most prevalent fields in the cancer patient population: pulmonary, urological and breast cancers (10 cases per field). Each case, presented in a short scenario, was followed by three related test items.

### Subjects

Three groups were sampled, representing three levels of clinical experience. The first group consisted of 4th-year medical students (n = 70) from the University of Montreal. Students took the exam immediately after a lecture given on radiation oncology. Only four of them possessed clinical experience in radiation oncology, acquired by an elective rotation (students taking these elective rotations often consider specializing in radio-oncology). The second group consisted of the population of residents of the three residency programs in radiation oncology in the province of Quebec (Montreal, Laval and McGill Universities) – a total of 52 residents. The third group consisted of the whole population of board-certified practitioners in radiation oncology of the province of Quebec (n = 62). The test was administered in the French language.

Each examinee received instructions on the particular format of the SCT and on the classification of the AJCC (American Joint Committee on Cancer) of the tested cancers. Participation was voluntary. Demographic data was collected for students and residents. Anonymity was guaranteed for certified board specialists. The project was approved by the ethics committee of the University of Montreal.

### Scoring

SCT scoring is based on the comparison of answers provided by examinees with those of a reference panel, composed of physicians with experience in the field. Panel members are asked to complete the test individually, and their answers are used to develop the scoring key [9]. In this study the radio-oncologists of the province of Quebec made both the third level of clinical experience and the panel of reference.

With this scoring method, the maximum score for each question is 1, for the modal answer from the reference panel. Other panel members' choices are attributed a partial credit, proportional to the number of members having provided that answer on the Likert scale divided by the modal value for the item. Answers not chosen by any panel members receive zero. For example, suppose the reference panel, made of 42 radio-oncologists, respond to a question in the following way: none choose the "+2", and "+1" ratings, 2 choose the "0" rating, 10 choose the "-1" rating and 30 choose the "-2" rating. The modal answer in this example is "-2". An examinee choosing this rating will receive 1. Selecting the "-1" rating will earn 0.33 (10/30) and the "0" rating, 0.06 (2/30). No points are accorded for selecting the "+1" or "+2" ratings.

With this method, all questions have the same maximum (1) and minimum (0) value. Scores obtained on each question are added to obtain a total score for the test. With SCTs, a theoretical score of 100 would mean that the person had answered each item in the same way that the majority of panel members did. In reality, such a score is never reached, even by panel members. Panel means found in tests administered in other specialities were generally in the 80s.

### Statistical analysis

To avoid bias, when radio-oncologists where used as members of the panel, scores for each question were computed using a scoring key that excluded their own response to that question. When they were studied as third level of experience, their scores were computed with the scoring key used for the other participants.

An item analysis was done to detect problematic questions. Questions with a low item-total correlation (r < 0.10) were removed. The normality of the distributions was evaluated with the Kolmogorov-Smirnov statistical test. The total scores of the subjects of each group were distributed. Reliability was estimated using the Cronbach alpha internal consistency coefficient.

The Levene test was used to evaluate the homogeneity of the variance of the three groups. ANOVA analysis was planned for group comparison. In case of lack of variance homogeneity, non-parametric alternatives were used. To evaluate the capacity to significantly discriminate the scores of the three groups, the non-parametric Kruskall-Wallis test was applied. The non-parametric Mann-Whitney test was used to assess more specifically the difference in scores between residents and radiation oncologists, junior residents (PGY-1 to PGY-3) and senior residents (PGY-4 to PGY-5).

All tests were bilateral, and *p *values < 0.05 were considered statistically significant. No correction for multiple tests was applied. The test results were treated anonymously. The analysis was done with the SPSS (Statistical Package for Social Sciences) software, version 11.0.

### Feasibility

Data were collected informally on test construction difficulties and on residents' and board-certified specialists' reactions to the content and format of the test.

## Results

### Subjects

Participants signed a consent form before taking the test. All 70 students agreed to participate. Among residents, all those from the University of Montreal (22), half of those from McGill (8/16) and 8 out of 14 from Laval took the test (no reason was provided by those who declined, but at McGill it was mainly a result of the language barrier). The 38 participating residents represent 72% of radiation oncology residents of the province, 70% (26) were juniors and 30% (11) were seniors (1 resident did not specify his/her year of residency). Forty-seven (76%) of the 62 board-certified radiation oncologists in the province agreed to participate. Among them, 81% had their practice in University hospitals. While there was no time constraint, most participants completed the test in under an hour.

Among board-certified radiation oncologists three were outliers (total test score under two standard deviations from the mean), and two had too many missing data. All five were taken out of the group. The panel and third level of experience was therefore made up of 42 persons.

### Missing data

One student and one resident were removed from the study due to too many missing data (more than four missing answers in either the pulmonary, urological or breast cancer sections). For all other participants, missing answers were replaced by the average score of all other questions from that section of the test. This represents less than 0.5% of all test answers. Participating in the analyses were 69 students, 37 radiation oncology residents and 42 radiation oncologists.

### Item analysis

The test taken by participants was composed of 10 cases in each section with three related questions. After item analysis, five questions were removed from the lung cancer section, five from the urological cancer section, and 10 from the breast cancer section. A maximum of two questions were discarded per case. After item optimization, the test comprised 30 cases and 70 questions. The normality of score distributions was verified with the Kolmogorov-Smirnov statistical test (Z > 0.558; p > 0.736).

### Reliability

The Cronbach alpha coefficient value for the optimized test is 0.90 (0.72 for the 25 questions on lung cancer, 0.78 for the 25 questions on urological cancer, and 0.78 for the 20 items on breast cancer).

### Participant scores

**Figure 2:**
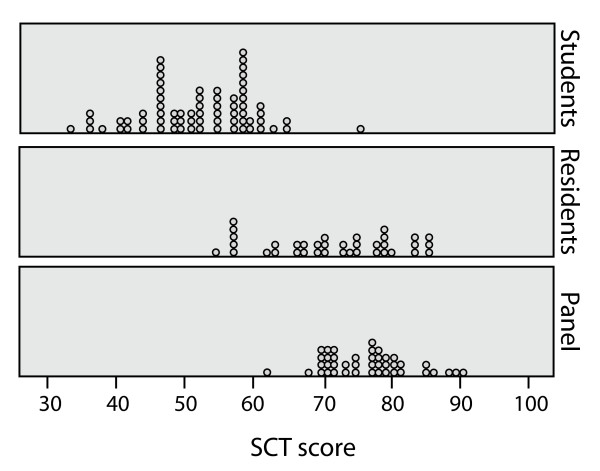
**SCT Score Distributions for Each Group**.

The mean score and the score variability are 51.6 (SD = 8.2; range 32.7–74.9) for students, 71.2 (SD = 9.5; range = 53.2–85.8) for residents, and 76.7 (SD = 6.1; range = 61.8–90.2) for board-certified radiation oncologists. The score distributions for each group are presented graphically in Figure 2. The score of one of the students (74.9) differs significantly from the average. This individual did a one-month rotation in radiation oncology and a one-month rotation in medical oncology.

Since the variances in the mean scores were not homogeneous (*p *= 0.016), non-parametric tests were used to evaluate the capacity of the test to detect differences according to clinical experience. There was a significant difference (*p *< 0.001) between the mean of the scores of the three groups of examinees. There is also a significant difference within residents, with junior residents (PGY-1 to PGY-3) having a mean of 68.9 ( ± 10.0) and senior residents (PGY-4 to PGY-5) of 76.5 ( ± 5.0; Z = -2.193, *p *= 0.028).

### Feasibility of and reactions to the test format

Despite their lack of knowledge and experience in the field, students were stimulated by the test format and were eager to get their test result. Students, residents and specialists completed the test with pleasure, and recruitment for the study was easy. Participants from the three groups succeeded in completing the test in a short time (less than an hour for most) and expressed their appreciation of the similarity between the situations described in the scenarios and real situations encountered in their practice.

## Discussion

With SCT, examinees are probed on a specific component of clinical reasoning: data interpretation, i.e. a crucial step within the clinical reasoning process [14]. It measures the degree of concordance between the examinee's performance and that of a reference panel on a series of case-based tasks. As it is inferred that high scores correspond to optimal use of information in the context of these specific tasks, the test therefore provides an indication of clinical reasoning quality. Clinicians find the test appealing because it contains cognitive tasks similar to those they encounter during their daily practice. Furthermore, as opposed to many other tests that require revision of knowledge for optimal performance, a clinician can fill out the test at any time without any preparation.

With SCT, examinees are not assessed against pre-set criteria or compared to their own group. Residents are physicians who wish to become, after training, members of the population of certified specialists of their field of study. Thus it is legitimate to compare their reasoning performance to a panel that is representative of physicians in that field. The panel, made up of 76% of board-certified radio-oncologists of the province, was highly representative of this population.

Test scores appear reliable, with Cronbach alpha coefficient reaching a value of 0.90 for 70 questions and for one hour of testing time. This compares very favourably with other test formats when compared in units of testing time [15]. The test showed a capacity to reflect clinical experience in the field. Participants with more experience in radiation oncology scored higher on the SCT. This was true for students when compared with the other two levels, for residents when compared with board-certified radiation oncologists and for junior residents when compared with senior residents. These results indicate that the SCT format should be useful for documenting learning alongside residency training.

Some of the results warrant comments. One student obtained a significantly higher score than the other members of his group. This student had completed two elective rotations in the field and was aiming for a residency in radiation oncology. Senior residents had scores that were close to those of the panel, thus indicating readiness for autonomous practice in the tested domains. On the other hand, some students and residents had low scores, indicating that SCT may potentially be used to identify residents who might not have good clinical judgment and may need to take remedial action.

The study has several limitations. It addresses only three specific areas of radiation oncology, and participants come from a limited geographic area. In the future, it would be interesting to repeat this experiment using a tool extended to include other pathologies in oncology (gynaecological, digestive, head and neck, etc). These spheres of competency have fewer practising experts and the results could be different. The majority of experts who completed the SCT practise in a university centre (81%) and often specialize in one or more specific areas of radiation oncology. Therefore, certain panel members who answered questions on lung cancer have not actually treated this pathology for many years. A forthcoming study will examine the influence of this specialization of radiation oncologists and the optimal number of panel members.

## Conclusion

SCT seems to measure a dimension of reasoning and knowledge that is different from those evaluated by usual assessment tools. It explores the interpretation of data in a clinical context, with ability clearly related to clinical experience. This study provides evidence in favour of SCT as a reliable and valid tool to evaluate the clinical reasoning of radiation oncology residents. The use of this instrument will allow for a more comprehensive evaluation of a resident's performance in this specialty. A low score on SCT could indicate residents who need assistance in the development of their reasoning capacity.

## Competing interests

The authors declare that they have no competing interests.

## Authors' contributions

CL contributed to conception and design, acquisition of data and interpretation of data and has been involved in drafting the manuscript. RG contributed to conception and design, analysis and interpretation of data and has been involved in revising the manuscript critically. DN contributed to acquisition of data and revised the manuscript. BC contributed to conception and design, analysis and interpretation of data and has been involved in drafting the manuscript. All authors read and approved the final manuscript.
